# Effect of one-lung ventilation on the correlation between left and right cerebral saturation

**DOI:** 10.1186/s12871-023-02001-7

**Published:** 2023-02-08

**Authors:** Cai-Juan Zhang, Jia-Hui Ma, Fan Jin, Xiu-Hua Li, Hui-Qun Jia, Dong-Liang Mu

**Affiliations:** 1 Department of Anesthesiology, Fourth Hospital of Hebei Medical University, Shijiazhuang, Hebei Province China; 2 Department of Anesthesiology, Peking University First Hospital, Xishiku Street No.8, Beijing, 100034 China; 3 Department of Anesthesiology, Tangshan Gongren Hospital, Hebei, China

**Keywords:** Cerebral tissue saturation, Correlation, One-lung ventilation, Lung cancer

## Abstract

**Background:**

To investigate if the correlation between left and right cerebral tissue oxygen saturation (SctO_2_) was affected by one-lung ventilation (OLV) in patients undergoing lung cancer surgery.

**Methods:**

Patients who underwent surgery for lung cancer were enrolled. Left and right SctO_2_ were collected during anesthesia. The primary outcome was the correlation between left and right SctO_2_ at 30 min after OLV which was analysed by Pearson correlation and linear regression model. Secondary outcomes included the trend of left–right SctO_2_ change over the first 30 min after OLV, correlation of left–right SctO_2_ during OLV for each patient; maximal difference between left–right SctO_2_ and its relationship with postoperative delirium.

**Results:**

Left–right SctO_2_ was moderately correlated at baseline (*r* = 0.690, *P* < 0.001) and poorly correlated at 30 min after OLV (*r* = 0.383, *P* < 0.001) in the Pearson correlation analysis. Linear regression analysis showed a poor correlation between left and right SctO_2_ at 30 min after OLV (*r* = 0.323, *P* < 0.001) after adjusting for confounders. The linear mixed model showed a change in left–right SctO_2_ over the first 30 min after OLV that was statistically significant (coefficient, -0.042; 95% CI, -0.070–-0.014; *P* = 0.004). For the left–right SctO_2_ correlation during OLV in each patient, 62.9% (78/124) patients showed a strong correlation, 19.4% (24/124) a medium correlation, and the rest a poor correlation. The maximal difference between the left and right SctO_2_ was 13.5 (9.0, 20.0). Multivariate analysis showed that it was not associated with delirium (odds ratio [OR], 1.023; 95% CI, 0.963–1.087; *P* = 0.463).

**Conclusions:**

The correlation between left and right SctO_2_ was affected by one-lung ventilation in patients undergoing lung cancer surgery. This result indicates the requirement of bilateral SctO_2_ monitoring to reflect brain oxygenation.

**Trial registration:**

This study was a secondary analysis of a cohort study approved by the Clinical Research Review Board of Peking University First Hospital (#2017–1378) and was registered in the Chinese Clinical Trial Registry on 10/09/2017 (http://www.chictr.org.cn, ChiCTR-ROC-17012627).

**Supplementary Information:**

The online version contains supplementary material available at 10.1186/s12871-023-02001-7.

## Background

Near-infrared spectroscopy (NIRS) has been widely used in patients undergoing non-cardiac surgery because it is a non-invasive, continuous, and timely method for monitoring regional cerebral tissue oxygen saturation (SctO_2_) [1]. The light source of the NIRS device emits a constant intensity of radiation in the near–infrared spectrum which can penetrate tissues and be absorbed by oxyhaemoglobin and deoxyhaemoglobin [2]. Sensors at a set distance receive a varied spectrum intensity to enable the calculation of the concentration of oxyhaemoglobin according to the Beer–Lambert law [2]. Both ipsilateral and bilateral sensors on the forehead have been used for SctO_2_ monitoring in different studies [3, 4]. One major concern is the discrepancy between left and right SctO_2_ readings. For example, a cohort study of healthy patients showed that the SctO_2_ of the ipsilateral sensor showed better accuracy with reference to jugular bulb venous oxygen saturation (SjO_2_) on the same side than on the contralateral side, and the comparison of readings between the two sides on a subject-by-subject basis showed a wide range of discrepancy [5]. This difference in bilateral readings has also been reported in pre-term infants [6].

Evidence in healthy volunteers showed that respiratory parameters significantly influence the accuracy of SctO_2_ measurement; for example, hypoxia or hypocapnia may increase measurement bias by approximately 10% [7, 8]. In clinical settings, respiratory parameters have also profound effects on cerebral saturation. First, the change of end-tidal carbon dioxide (EtCO_2_) is highly associated with SctO_2_. A cohort study of 20 surgical patients found that mild hypercapnia (approximately 45 mmHg) significantly increased SctO_2_ (68% vs. 55% in the normocapnia group) [9]. In contrast, hypocapnia (i.e., induced by hyperventilation) led to an approximately 10% decrease in SctO_2_ in patients undergoing non-neurosurgical procedures [10]. Second, lower arterial oxygen pressure is associated with a higher risk of cerebral desaturation [11]. In contrast, a higher inspired oxygen fraction is useful for the elevation of SctO_2_ in patients undergoing carotid endarterectomy [12].

One-lung ventilation (OLV) is a necessary technique in thoracic surgery. The incidences of hypoxia and hypercapnia are as high as 50% during OLV [4, 13]. In a cohort study of 15 patients undergoing OLV, hypercapnia (arterial partial pressure of carbon dioxide = 50 mmHg) increased SjO_2_ from 54 to 69% [14]. However, there is limited data to illustrate whether OLV affects the agreement between left and right SctO_2_ and if the change in difference between left–right readings has clinical significance.

The present study was primarily designed to investigate whether OLV influences the agreement between the left and right SctO_2_ readings.

## Methods

### Ethic and registration

This study was a secondary analysis of a cohort study which was approved by the Clinical Research Review Board of Peking University First Hospital (#2017–1378) and registered in the Chinese Clinical Trial Registry (http://www.chictr.org.cn, ChiCTR-ROC-17012627) [4]. Written informed consent was obtained from all subjects and/or their legal guardians. All methods were performed in accordance with the relevant guidelines and regulations at Peking University First Hospital and Fourth Hospital of Hebei Medical University.

### Patient inclusion and exclusion criteria

Adult patients (age ≥ 55-year-old) who received OLV under general anesthesia with or without epidural or paravertebral block were enrolled. Patients were excluded if they met any of the following criteria: 1) no record of SctO_2_ or 2) no record of EtCO_2_ or peripheral pulse oxygenation (SpO_2_).

### Primary outcome

The primary outcome was agreement of left–right SctO_2_ readings at 30 min after OLV. SctO_2_ was monitored at the bilateral forehead using the FORE-SIGHT ELITE tissue oximeter (CASMED, Branford, CT, USA) from baseline to the end of surgery [4]. The readings of SctO_2_ were generated by oximetry every 2 s and extracted from the monitor at the end of surgery. Left–right SctO_2_ readings were collected in pairs at 1 min intervals.

Baseline measurements were obtained before anesthesia induction, with the patients resting and breathing room air. The screen of the tissue oximeter was covered with an opaque bag to blind the anesthesia providers for monitoring. Dedicated research personnel checked the tissue oximeter every 10 min to ensure proper function.

### Secondary outcomes

Secondary outcomes included the correlation of left–right SctO_2_ at eight time points before, during, and after OLV, the trend of left–right SctO_2_ over the first 30 min after OLV, the agreement of left–right SctO_2_ during OLV for each patient, the maximal difference between left–right SctO_2_, and its relationship with postoperative delirium.

Correlations of left–right ScO_2_ at eight time points were calculated at baseline, at 5 min interval during the first 30 min after the beginning of OLV, and at 5 min interval during the first 15 min after the end of OLV. The trends of left–right SctO_2_ over the first 30 min after OLV were analysed to investigate whether they had the same trends of change. Left–right SctO_2_ readings during OLV in each patient were collected in pairs, and their agreements were analysed individually. The maximal difference was defined as the maximal value between each pair of left–right readings, and its relationship with delirium was analysed accordingly.

Delirium was assessed twice daily (at 06:00–08:00 and 18:00–20:00) within postoperative 5 days using the Chinese version of the Confusion Assessment Method (CAM) in non-intubated patients and the CAM for intensive care unit (CAM-ICU) in intubated patients [4, 15–17]. The researchers who were responsible for delirium assessment participated in a 4 h training session and were not allowed to access patient data during the research [16, 17].

### Anesthesia and perioperative care

All patients underwent the same monitoring on arrival at the operating room, including SpO_2_, electrocardiography, and non-invasive blood pressure. Bispectral Index (BIS), EtCO_2_, and nasopharyngeal temperature were monitored during general anesthesia which have been described in the original study as previously reported [4]. Invasive arterial blood pressure and central venous pressure can be used when necessary.

Anesthesia was induced using propofol (2–4 mg/kg) and sufentanil (site-effect concentration, 0.2*–*0.5 ng/ml). Anesthesia maintenance was administrated by propofol (4*–*10 mg/kg/h) and sufentanil (site-effect concentration, 0.2*–*0.5 ng/ml) via continuous infusion. Anesthesia depth was maintained a BIS value between 40 and 60 as previously reported [4].

A double-lumen endotracheal tube was used for intubation. The tidal volume was set at 6*–*8 ml/kg. The aim of the minute ventilation volume was to maintain EtCO_2_ at 35*–*45 mmHg and SpO_2_ ≥ 92%. Fluctuation in blood pressure was maintained within 20% of the baseline value. Nasopharyngeal temperature was maintained at 36*–*37 °C.

Postoperative pain was assessed using numeric rating scale (an 11-point scale where 0 indicates no pain and 10 indicates the worst pain). Patient-controlled intravenous analgesia (PCIA) was used to maintain a pain score of 3 or lower. The program of PCIA was set to deliver a background infusion of sufentanil at 1.25 μg/h and a 2.5 μg bolus with a lock-out interval of 8 min for breakthrough pain [4].

### Statistical analysis

#### Power calculation

The Pearson’s correlation coefficient of left–right SctO_2_ at baseline was 0.690, and that at 30 min after OLV was 0.383. If the significance level was set at 0.05, the present sample size of 124 patients yielded a power of 0.99.

#### Outcome analysis

Continuous variables with normality are presented as mean (standard deviation, SD) and variables without normality are presented as median (interquartile range, IQR). The binary variables are presented as numbers (percentages).

For the primary outcome, the correlation between left–right SctO_2_ at 30 min after OLV was first analysed using Pearson correlation. A linear regression model was then employed to investigate the correlation of left–right SctO_2_ after adjusting for confounders. Selection of confounders was based on the results of previous trials, including comorbidities (age, diabetes, and hypertension) and parameters at 30 min after OLV (i.e., SpO_2_, EtCO_2_, mean arterial blood pressure) [7, 8, 18–21].

For the secondary analysis, the correlation of left–right SctO_2_ at fixed time points was also analysed in line with the primary outcome. The Bonferroni method was used to control for type I errors. A linear mixed model was used to compare the trend of left–right SctO_2_ over the first 30 min after OLV, with adjustment for confounders. Pearson correlation was used to analyse the agreement between left and right SctO_2_ during OLV in each patient. The Pearson’s correlation coefficients were divided into three groups: strong (*r* ≥ 0.7), medium (0.5 ≤ r < 0.7), and weak (*r* < 0.5) correlations. The maximal differences are presented as medians (IQR). Its relationship with postoperative delirium was analysed using multivariate logistic analysis after adjusting for confounders. Confounders were first selected by univariate analysis, and variables with *P* < 0.05 were entered into multivariate analysis.

Statistical significance was set at *P* < 0.05. Analyses were performed using R 3.6.0 (R Foundation for Statistical Computing, Vienna, Austria, 2019).

## Results

### Patients

**Fig. 1:**
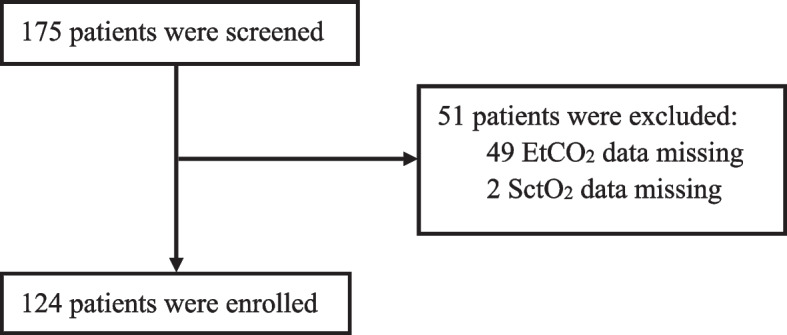
Flowchart. Flowchart of patient enrolment. EtCO_2_ = end-tidal carbon dioxide; SctO_2_ = cerebral tissue oxygen saturation

A total of 124 patients were enrolled in the present study (Fig. 1). Mean age of enrolled patients was 64.7 ± 6.7 years old (Table 1). A total of 34 (27.5%) patients received epidural anesthesia or PVB for intraoperative analgesia. The median duration of OLV was 3.1 ± 1.5 h. The overall trend of the left and right SctO_2_ during anesthesia is described in Additional file 1.

**Table 1:** Baseline characteristics

Variables	Total (*n* = 124)
Age, mean ± SD, year	64.7 ± 6.7
Female, n (%)	64 (51.6)
BMI, mean ± SD, kg/m^2^	24.9 ± 3.4
Smoking, n (%) ^a^	21 (16.9)
Preoperative comorbidity, n (%)	
Hypertension	55 (44.4)
Diabetes	20 (16.1)
Coronary artery disease	16 (12.9)
Stroke	9 (7.3)
Arrhythmia	10 (8.1)
COPD	4 (3.2)
Asthma	2 (1.6)
Hyperlipidemia	3 (2.4)
Preoperative MoCA score, median (IQR)	26 (23, 28)
ASA classification, n (%)	
I	1 (0.8)
II	100 (80.6)
III	23 (18.5)
Site of surgery, n (%)	
Left lung	49 (39.5)
Right lung	75 (60.5)
Surgery type, n (%)	
Lobectomy	123 (99.2)
Pneumonectomy	1 (0.8)
Intraoperative drugs	
Sufentanil, μg, median (IQR)	40.0 (30.0, 73.9)
Propofol, mg, median (IQR)	809.5 (200.0, 1268.5)
Nitrous oxide, n (%)	51 (41.1)
Sevoflurane, n (%)	57 (46.0)
Midazolam, n (%)	33 (26.6)
Anesthesia type, n (%)	
GA only	90 (72.6)
GA + epidural anesthesia	10 (8.1)
GA + paravertebral block	24 (19.4)
Intraoperative hypotension, n (%) ^b^	55 (44.4)
Intraoperative hypoxia, n (%)	14 (11.3)
Duration of OLV, mean ± SD, h	3.1 ± 1.5
Duration of surgery, mean ± SD, h	3.4 ± 1.6
Duration of anesthesia, mean ± SD, h	4.4 ± 1.7
Pain score at first day after surgery, NRS, median (IQR) ^c^	4 (3, 5)
Postoperative delirium, n (%)	25 (20.2)

*SD* Standard deviation, *BMI* Body mass index, *COPD* Chronic Obstructive pulmonary disease, *MoCA* Montreal cognitive assessment, *IQR* Interquartile range, *ASA* American Society of Anesthesiology, *GA* General anesthesia, *OLV* One-lung ventilation, *NRS* Numeric rating score

^a^ Patients were categorized as a smoker if the smoking index (smoking index = cigarettes per day × year of tobacco use) was > 400

^b^ Hypotension was defined as systolic blood pressure < 90 mmHg or 70% of the baseline value that required treatments

^c^ The severity of pain during movement was assessed using a numeric rating scale, i.e., an 11-point score scale where 0 indicates no pain and 10 indicates the worst pain

### Correlation of baseline left and right SctO_2_

**Fig. 2:**
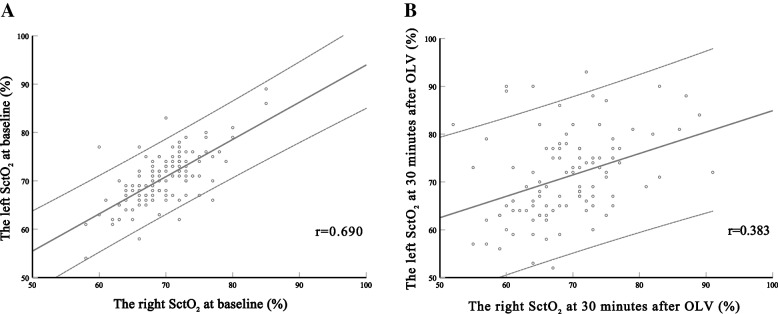
Correlation of left–right SctO_2_ at baseline and 30 min after one-lung ventilation. Pearson correlation analysis showed medium correlation between left and right cerebral tissue oxygenation (**A**, *r* = 0.690, *P* < 0.001) at baseline and poor correlation at 30 min after one-lung ventilation (B, *r* = 0.383, *P* < 0.001)

Baseline left and right SctO_2_ were 70.5 ± 5.4 and 69.5 ± 4.8 respectively. The Pearson’s correlation coefficient between the baseline left and right SctO_2_ was 0.690 (*P* < 0.001) (Fig. 2A). After adjusting for confounders, linear regression analysis showed that the Pearson’s correlation coefficient between them was 0.805 (*P* < 0.001).

### Primary outcome

At 30 min after OLV, the values of left and right SctO_2_ were 70.9 ± 8.9 and 68.9 ± 7.4 respectively. The Pearson’s correlation coefficient of left and right SctO_2_ was 0.383 (*P* < 0.001) (Fig. 2B). After adjusting for confounders, linear regression analysis showed that the Pearson’s correlation coefficient between the left and right SctO_2_ was 0.323 (*P* < 0.001) (Table 2).

**Table 2:** Pearson’s correlation coefficients between bilateral SctO_2_ at fixed time points

**Variables**	**Baseline**	**Minutes after OLV start**				**Minutes after OLV end**		
	**T0**	**T1**	**T2**	**T3**	**T4**	**T5**	**T6**	**T7**
		**5 min**	**10 min**	**15 min**	**30 min**	**5 min**	**10 min**	**15 min**
Left SctO_2_, % Mean ± SD	70.5 ± 5.4	73.7 ± 8.4	72.9 ± 8.3	72.0 ± 8.7	70.9 ± 8.9	73.4 ± 8.2	73.6 ± 8.0	73.6 ± 8.3
Right SctO_2_, %, Mean ± SD	69.5 ± 4.8	73.2 ± 8.2	71.2 ± 7.4	70.4 ± 7.5	68.9 ± 7.4	72.0 ± 7.1	72.6 ± 7.1	73.1 ± 7.0
Pearson’s correlation coefficient ^a^	0.690	0.385	0.408	0.414	0.383	0.361	0.359	0.422
P	**< 0.001**	**< 0.001**	**< 0.001**	**< 0.001**	**< 0.001**	**< 0.001**	**< 0.001**	**< 0.001**
Linear regression analysis coefficient ^b^	0.805	0.391	0.371	0.378	0.323	0.361	0.246	0.368
P	**< 0.001**	**< 0.001**	**< 0.001**	**< 0.001**	**0.001**	**< 0.001**	0.016	**< 0.001**

*SctO*_*2*_ Cerebral tissue oxygen saturation, *SD* Standard deviation, *IQR* Interquartile range, *OLV* One-lung ventilation

^a^ Pearson correlation analysis was firstly used to investigate the relationship between left and right cerebral tissue oxygenation

^b^ Linear regression analysis was used to investigate the correlation between bilateral SctO_2_ after adjustment for comorbidities (age, diabetes, hypertension) and parameters at measurements (mean arterial blood pressure, peripheral pulse oxygenation, and end-tidal carbon dioxide)

### Secondary outcomes

#### Correlations between left–right SctO_2_ at fixed time points

Correlations between the left and right SctO_2_ at predefined time points are presented in Table 2. At each point, the left–right SctO_2_ was poorly correlated, even 15 min after the end of OLV (*r* = 0.368, adjusted *P* < 0.001).

#### The trend of SctO_2_ changes during the first 30 min after OLV

**Fig. 3:**
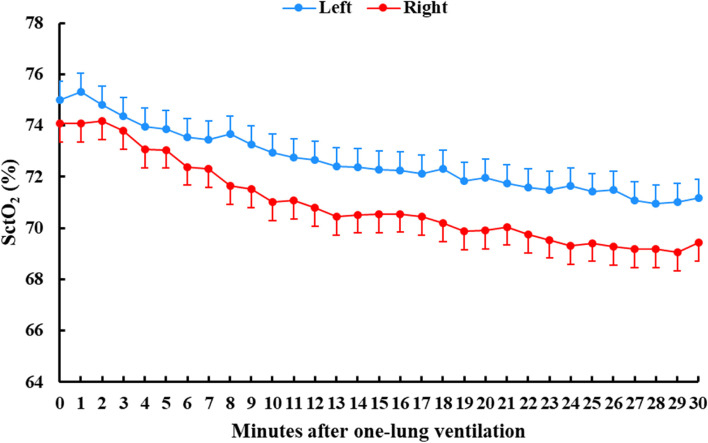
Comparison of left and right SctO_2_ trend over the first 30 min after one-lung ventilation. Linear mixed model showed that the change of left and right SctO_2_ trend over the first 30 min after one-lung ventilation presented statistical significance (coefficient, -0.042; 95% CI, -0.070–-0.014; *P* = 0.004). The dot indicated the least square means of SctO_2_ and the dash indicated its SD

The trend of left and right SctO_2_ changes during the first 30 min after OLV is depicted with the least square means (LSD) in Fig. 3. The linear mixed model showed that the change in SctO_2_ over time was statistically significant between the two sides (coefficient, -0.042; 95% CI, -0.070–-0.014; *P* = 0.004).

#### Correlation between left and right SctO_2_ in each patient

**Fig. 4:**
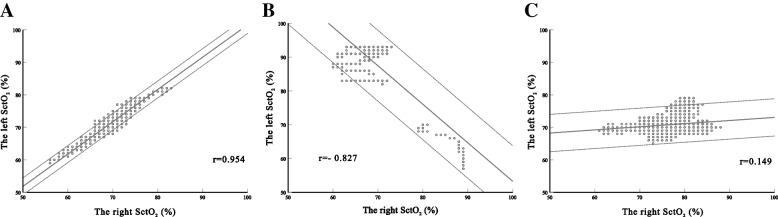
Samples of correlation between left and right SctO_2_ in each patient. Correlation of left and right SctO_2_ during OLV in each patient was individually analysed using Pearson correlation analysis. Here, there are three samples which showed the uncertain relationship on a subject-to-subject type

The correlation of left–right SctO_2_ in each patient is presented in Additional file 2. In these patients, 62.9% (78/124) showed a strong correlation, 19.4% (24/124) showed a medium correlation, and 17.7% (22/124) showed a poor correlation. We provided three samples of correlation plots which indicated that the correlation between the left and right varied on a subject-by-subject basis (Fig. 4).

#### Maximal difference and its relationship with postoperative delirium

The maximal difference between the left–right readings during OLV was 13.5 (9.0, 20.0). In the multivariate analysis, it was not associated with postoperative delirium (OR, 1.023; 95% CI, 0.963–1.087; *P* = 0.463) after adjusting for intraoperative hypoxia, hypotension, and use of midazolam (Table 3).

**Table 3:** The association between the absolute difference of left and right SctO_2_ and postoperative delirium

Variables	Univariate analysis		Multivariate analysis	
	**Odds ratio (95% CI)**	**P**	**Odds ratio (95% CI)**	**P**
Max absolute difference (per 1% increase)	1.029 (0.976, 1.084)	0.286	1.023 (0.963, 1.087)	0.463
Intraoperative hypoxia (yes)	3.592 (1.117, 11.554)	**0.032**	3.647 (0.988, 13.457)	0.052
Intraoperative hypotension (yes)	2.735 (1.100, 6.800)	**0.030**	1.681 (0.608, 4.646)	0.316
Midazolam (yes)	2.750 (1.095, 6.907)	**0.031**	2.687 (0.951, 7.594)	0.062

A hypoxia was defined as peripheral oxygen saturation (SpO_2_) was lower than 90% and last 1 min

Hypotension was defined as systolic blood pressure < 90 mmHg or 70% of the baseline value that required treatment

*SctO*_*2*_ Cerebral tissue saturation, *CI* Confidence interval

## Discussion

The present study found that OLV increased the discrepancy in left–right SctO_2_ readings in patients undergoing thoracic surgery. The maximal difference in the left–right SctO_2_ was not associated with delirium.

Although there are data to support the poor correlation of bilateral SctO_2_ readings in healthy volunteers, the present study is the first to investigate this phenomenon in patients undergoing OLV [5]. It is obvious that the correlation between left and right SctO_2_ became poor at 30 min after OLV in comparison with baseline. We selected the correlation of left–right SctO_2_ at 30 min after OLV as the primary outcome because patients experience the most severe changes in respiratory parameters during this period. For example, the overall incidence of hypoxia during OLV was 58.1% (72/124), while 52.8% (38/72) occurred in the first 30 min after OLV.

One strength of our study is that we employed a linear regression model to adjust for confounders that might affect SctO_2_ measurement. Most studies only used simple correlation analysis, such as Pearson correlation, to compare bilateral SctO_2_ [5, 7]. This method is practical for healthy volunteers but is not suitable for patients in clinical settings. Age, diabetes, hypertension, SpO_2_, EtCO_2_, and mean arterial blood pressure may significantly affect the accuracy of SctO_2_ readings [7, 8, 18–21]. Our results showed that the correlation between left and right SctO_2_ at 30 min after OLV decreased from 0.383 to 0.323 after adjustment. This indicated that the interpretation of SctO_2_ during OLV should consider preoperative comorbidities and respiratory parameters.

Because the correlation at a single time point could not reflect the trend of change, we provided three methods to illustrate the question. First, we used a linear mixed model to compare the trend of SctO_2_ change during the first 30 min after OLV. Unlike classic correlation that measures the agreement between two variables, a linear mixed model can provide an analysis of the trend of repeated measurements [22]. Our results showed that the change trend between left–right SctO_2_ presented statistically significant over time. Second, we provided Pearson’s correlation coefficients at eight fixed time points as representative of changes before, during, and after OLV. Our results showed that the phenomenon of poor correlation between left and right SctO_2_ still existed at 15 min after the end of OLV. Third, we compared the correlation of left–right SctO_2_ during OLV for each patient with the subject-to-subject purpose. Our results showed that a strong correlation existed only in 62.9% of patients. An inverse correlation was observed in some patients.

Another interesting finding was that the maximal difference between the left and right SctO_2_ readings was approximately 13.5 which might have statistical significance for diagnosing cerebral desaturation. Our previous analysis showed that a relative decrease of 10% from the baseline SctO_2_ value was highly associated with delirium [4]. Taking this criterion as a reference, the incidence of cerebral desaturation was 61.5% on the left side and 57.5% on the right side. However, multivariate analysis showed that the maximal difference in left–right SctO_2_ was not related to postoperative delirium. The clinical significance of this difference warrants further investigation.

The discrepancy in the left–right SctO_2_ readings may be mainly attributed to two aspects. First, blood supply to the left and right hemispheres is generated from different arteries. Cerebrovascular disease, such as severe stenosis of the internal carotid artery and circle of Willis may impair oxygen supply and consumption on the ipsilateral side [23–25]. One limitation of the present study was that we did not perform preoperative ultrasound screening of the carotid artery; however, all patients underwent thorough physical examination, including auscultation of the carotid bruit and no positive events were reported in the medical records. Second, measurement bias may be attributed to the calculation algorithm. The readings of SctO_2_ are based on a calculation algorithm with a fixed ratio of cerebral mixed venous and arterial blood (i.e., 30:70 claimed by CASMED) [7]. Many factors induce variations in the in vivo ratio. For example, two studies reported that artificial hypoxia or hypocapnia may increase measurement bias in approximately 10% of healthy volunteers [7, 8]. In our study, we could not evaluate the accuracy of the left and right readings, because SjvO_2_ was not measured in the present study which was considered as a “gold” reference to calibrate SctO_2_.

Our results show that bilateral SctO_2_ at baseline is well correlated, but the trend of bilateral SctO_2_ is different and is significantly affected by OLV. This result suggests the application of bilateral SctO_2_ in clinical practice and studies. Further studies are needed to illustrate how to clinically act if a patient suffers from one or two-sided cerebral desaturation.

One limitation of the present study is its secondary analysis design. However, all these data were prospectively collected in a previous study which ensured the quality of the data. Second, the sample size was limited to 124 patients. Based on the results, the sample size yielded a statistical power of 0.99. Third, as discussed above, SjvO_2_ and carotid artery ultrasound were not performed.

## Conclusions

In the present study, we found that the correlation between left and right SctO_2_ was affected by OLV in patients undergoing thoracic surgery. This result indicates the requirement of bilateral SctO_2_ monitoring to reflect brain oxygenation. Further studies are needed to investigate whether this difference affects the patient outcomes.

## Supplementary Information

**Additional file 1.** Overall trend of left and right cerebral tissue oxygenation during anesthesia. Cerebral tissue oxygenation (%) per minute. The mean and SD of the left side are illustrated by the dodger blue line and light blue area, respectively. The mean and SD of the right side are shown by the red line and light red area, respectively. The time points were analysed when ≥ 10 patients were included.

**Additional file 2.** A total of 124 patients were included in the analysis for the calculation of Pearson’s correlation coefficient and *P* value during the first 30 minutes after OLV.

## Data Availability

The datasets generated and analysed during the current study are not publicly available due to institutional restrictions, but are available from the corresponding author upon reasonable request.

## Abbreviations

SctO_2_: Cerebral tissue oxygen saturation
OLV: One-lung ventilation
NIRS: Near-infrared spectroscopy
SjO_2_: Jugular bulb venous oxygen saturation
EtCO_2_: End-tidal carbon dioxide
SpO_2_: Peripheral pulse oxygenation
CAM: Confusion Assessment Method
CAM-ICU: Confusion Assessment Method for intensive care unit
BIS: Bispectral Index
PCIA: Patient-controlled intravenous analgesia
