# Supplementary material for: Effect of one-lung ventilation on the correlation between left and right cerebral saturation
Source: BMC Anesthesiol. 2023 Feb 8;23:50. doi: 10.1186/s12871-023-02001-7 (PMC9906862; doi:10.1186/s12871-023-02001-7)

Additional file 1 Overall trend of left and right cerebral tissue oxygenation during anesthesia

Additional file 1 legend Cerebral tissue oxygenation (%) per minute. The mean and SD of the left side are illustrated by the dodger blue line and light blue area, respectively. The mean and SD of the right side are shown by the red line and light red area, respectively. The time points were analysed when ≥ 10 patients were included.

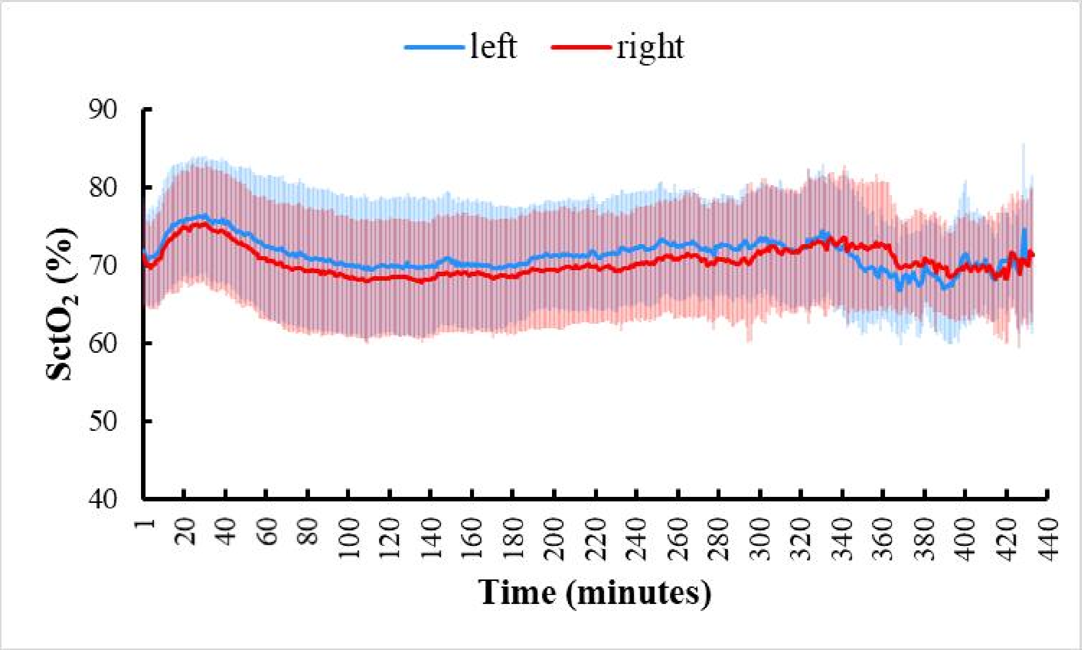

Supplement: Supplementary file 1 — Additional file 1. Overall trend of left and right cerebral tissue oxygenation during anesthesia. Cerebral tissue oxygenation (%) per minute. The mean and SD of the left side are illustrated by the dodger blue line and light blue area, respectively. The mean and SD of the right side are shown by the red line and light red area, respectively. The time points were analysed when ≥ 10 patients were included. [file 12871_2023_2001_MOESM1_ESM.pdf]
