# Supplementary material for: Effect of one-lung ventilation on the correlation between left and right cerebral saturation
Source: BMC Anesthesiol. 2023 Feb 8;23:50. doi: 10.1186/s12871-023-02001-7 (PMC9906862; doi:10.1186/s12871-023-02001-7)
Supplement: Supplementary file 2 — Additional file 2. A total of 124 patients were included in the analysis for the calculation of Pearson’s correlation coefficient and P value during the first 30 minutes after OLV. [file 12871_2023_2001_MOESM2_ESM.pdf]

Additional file 2 A total of 124 patients were included in the analysis for the calculation of Pearson's correlation coefficient and P value during the first 30 minutes after OLV.

**Case number**	**r**	**P**
1	0.431	<0.001
2	0.778	<0.001
3	0.960	<0.001
4	0.603	<0.001
5	0.943	<0.001
6	0.874	<0.001
7	0.621	<0.001
8	0.219	<0.001
9	0.909	<0.001
10	0.736	<0.001
11	0.842	<0.001
12	0.545	<0.001
13	0.739	<0.001
14	0.697	<0.001
15	0.825	<0.001
16	0.443	<0.001
17	0.592	<0.001
18	0.232	<0.001
19	0.802	<0.001
20	0.513	<0.001
21	0.375	<0.001
22	0.792	<0.001
23	-0.189	<0.001
24	0.764	<0.001
25	0.851	<0.001
26	0.837	<0.001
27	0.727	<0.001
28	0.957	<0.001
29	0.860	<0.001
30	0.329	<0.001
31	0.767	<0.001
32	0.658	<0.001
33	0.698	<0.001
34	0.910	<0.001
35	0.954	<0.001
36	0.680	<0.001
37	0.909	<0.001
38	0.517	<0.001
39	0.665	<0.001
40	0.710	<0.001

41	0.820	<0.001
42	0.627	<0.001
43	0.867	<0.001
44	0.375	<0.001
45	0.908	<0.001
46	0.617	<0.001
47	0.955	<0.001
48	0.810	<0.001
49	-0.175	<0.001
50	0.891	<0.001
51	0.534	<0.001
52	0.794	<0.001
53	0.978	<0.001
54	0.938	<0.001
55	0.950	<0.001
56	0.907	<0.001
57	0.946	<0.001
58	0.816	<0.001
59	0.905	<0.001
60	0.959	<0.001
61	0.944	<0.001
62	0.987	<0.001
63	0.306	<0.001
64	0.928	<0.001
65	0.595	<0.001
66	0.751	<0.001
67	-0.096	<0.001
68	0.583	<0.001
69	0.944	<0.001
70	0.816	<0.001
71	0.664	<0.001
72	0.951	<0.001
73	0.104	<0.001
74	0.628	<0.001
75	0.855	<0.001
76	0.863	<0.001
77	0.844	<0.001
78	0.535	<0.001
79	0.491	<0.001
80	0.772	<0.001
81	0.647	<0.001
82	0.648	<0.001
83	0.500	<0.001
84	0.941	<0.001

85	0.984	<0.001
86	0.211	<0.001
87	0.929	<0.001
88	0.702	<0.001
89	0.962	<0.001
90	-0.827	<0.001
91	0.560	<0.001
92	0.149	<0.001
93	0.831	<0.001
94	-0.493	<0.001
95	0.975	<0.001
96	0.901	<0.001
97	0.973	<0.001
98	0.812	<0.001
99	0.798	<0.001
100	0.499	<0.001
101	0.846	<0.001
102	0.963	<0.001
103	-0.157	<0.001
104	0.797	<0.001
105	0.943	<0.001
106	0.859	<0.001
107	0.969	<0.001
108	0.918	<0.001
109	0.916	<0.001
110	0.909	<0.001
111	0.158	<0.001
112	0.429	<0.001
113	0.914	<0.001
114	0.699	<0.001
115	-0.075	<0.001
116	0.238	<0.001
117	0.816	<0.001
118	0.977	<0.001
119	0.976	<0.001
120	0.935	<0.001
121	0.868	<0.001
122	0.926	<0.001
123	0.888	<0.001
124	0.923	<0.001
